# Supplementary material for: How Does a Community Respond to Changes in Aircraft Noise? A Comparison of Two Surveys Conducted 11 Years Apart in Ho Chi Minh City
Source: Int J Environ Res Public Health. 2021 Apr 19;18(8):4307. doi: 10.3390/ijerph18084307 (PMC8074233; doi:10.3390/ijerph18084307)
Supplement: Supplementary file 1 [file ijerph-18-04307-s001.zip › ijerph-1170946-supplementary.pdf]

Supplementary material

How Does a Community Respond to Changes in Aircraft Noise? A Comparison of Two Surveys Conducted 11 Years apart in Ho Chi Minh City

Table S1. Similarities and differences in the methods of the two surveys.

Item	2008 Survey	2019 Survey
Noise source type	military and civil aircraft	civil aircraft
Exposure assessment methods	field measurements	estimated using noise map
Noise level at participants' residence	one exposure value per survey site	exact values at the residents' addresses
Choice of survey areas	selected to reflect exposure situation from minimum to loudest aircraft noise level	same as 2008 survey
Noise annoyance question and scale	ICBEN7's standardized question and scale	same as 2008 survey
Sleep quality question and scale	"What is the status of your daily sleep?"<br><br>a five-point scale (1. Extremely good; 2. Good; 3. Neutral; 4. Bad; 5. Extremely bad)	"During the past four weeks, how would you rate the quality of your sleep?"<br><br>a five-point scale (1. Very good; 2. Fairly good; 3. Fairly bad; 4. Very bad)

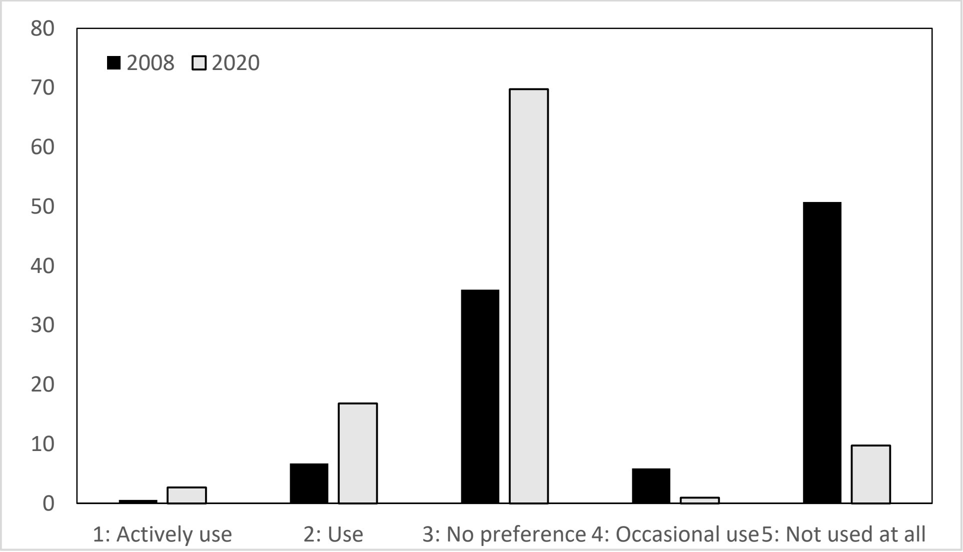

(a) Frequency of air-transport use

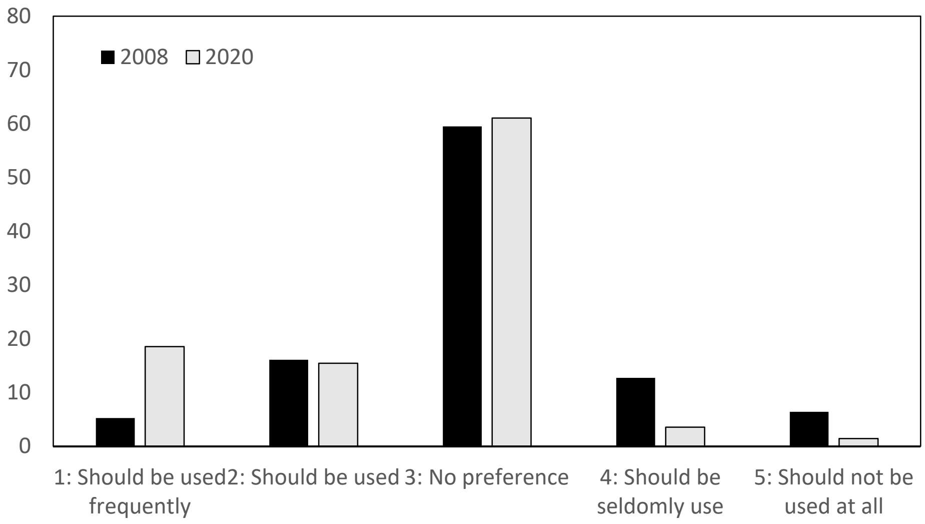

(b) Benefits to the society of air transport

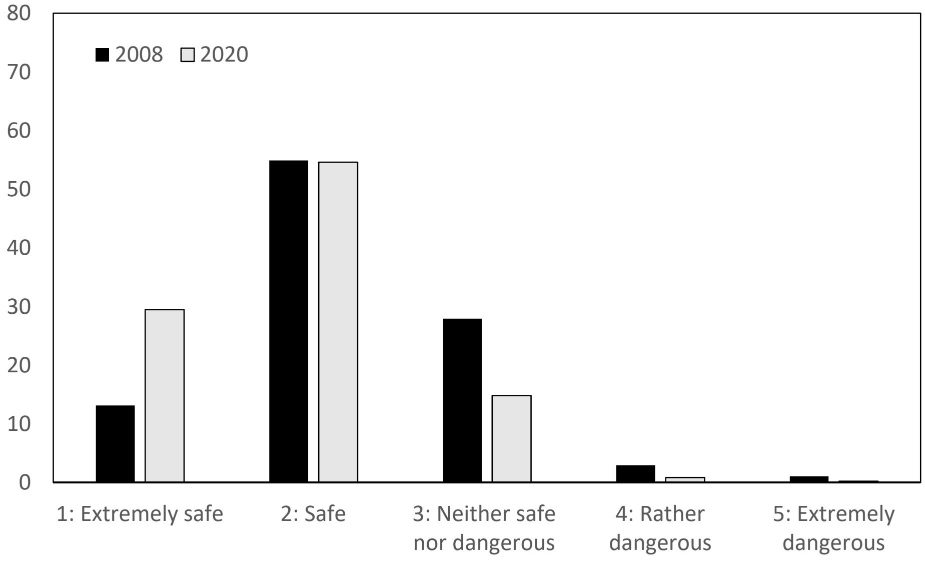

(c) Safety of air transport.

Figure S1. Comparison of the attitude to air transport as per the 2008 and 2020 surveys.
